# Supplementary material for: Salmonella Typhimurium with Eight Tandem Copies of blaNDM-1 on a HI2 Plasmid
Source: Microorganisms. 2023 Dec 22;12(1):20. doi: 10.3390/microorganisms12010020 (PMC10819877; doi:10.3390/microorganisms12010020)
Supplement: Supplementary file 1 [file microorganisms-12-00020-s001.zip › microorganisms-2703210-supplementary.pdf]

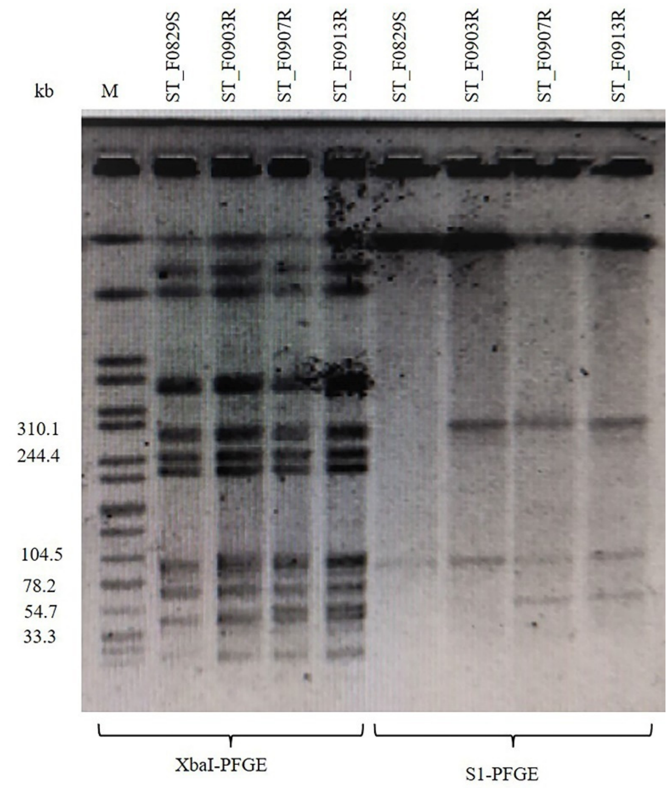

Supplementary Figure S1. The band profiles of XbaI-PFGE and S1-PFGE. Lane M, *Salmonella* serotype Braenderup strain H9812 as a molecular marker.

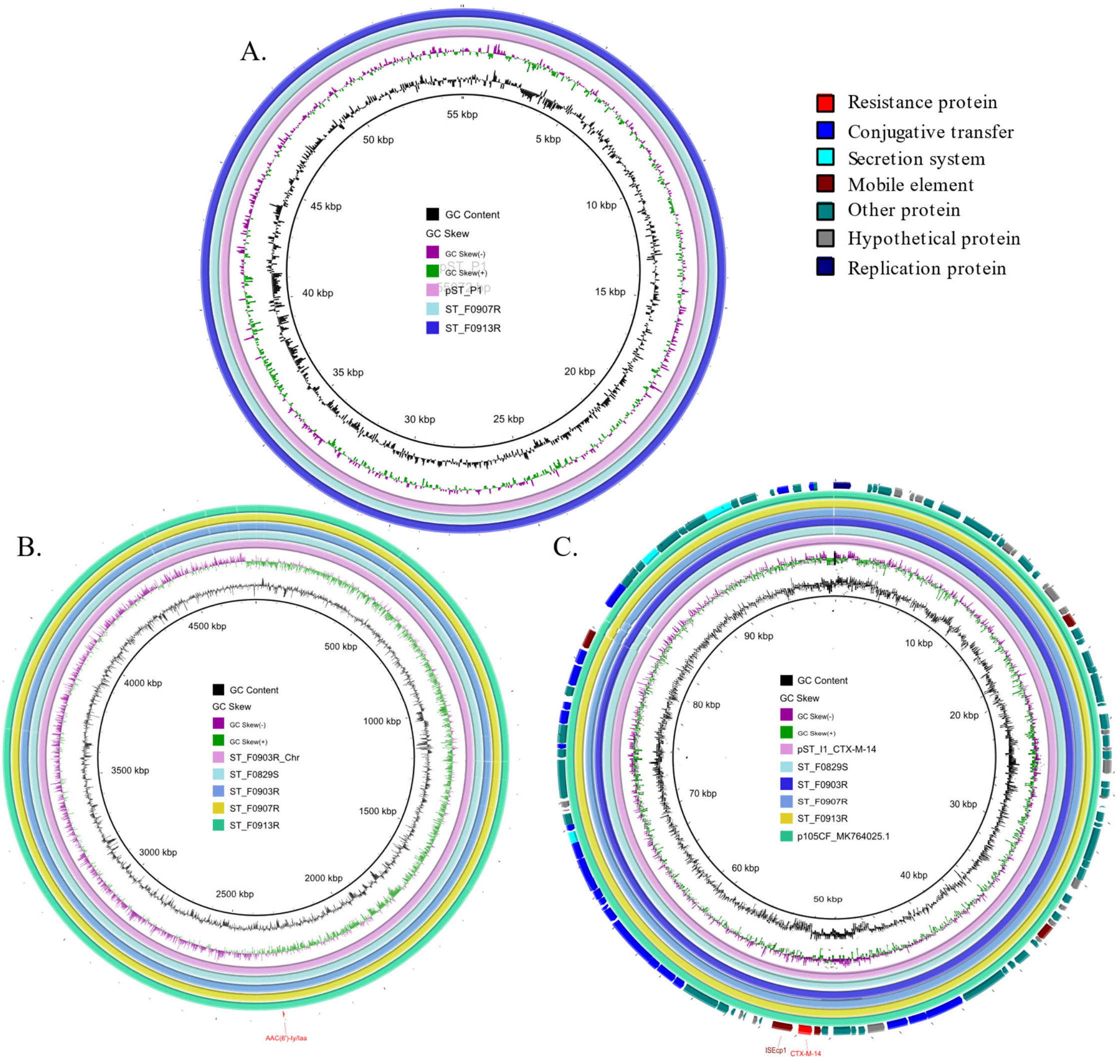

Supplementary Figure S2. Circular maps of chromosome and plasmids in the *Salmonella* Typhimurium isolates. A. The circle genome alignment map of pST_P1. The inner scale is marked in kbp. Circles range from 1 (the inner circle) to 5 (the outer circle). Circle 1, GC content, inward indicates lower than the average GC content, and outward indicates higher than the average GC content; circle 2, GC skew (G−C/G+C), values > 0 are in green, and values < 0 are in purple; circle 3, the complete sequence of pST_P1; circle 4−5, the draft genomes of *Salmonella* Typhimurium isolates. B. Comparative genome

analysis with the ST_F0903R chromosome as the reference sequence. Circles range from 1 (the inner circle) to 7 (the outer circle). Circle 3, the ST_F0903R strain chromosome sequence; circle 4-7, the draft genomes of *Salmonella* Typhimurium isolates. C. The circle genome alignment map of pST_I1_CTX-M-14. Circles range from 1 (the inner circle) to 9 (the outer circle). Circle 4−7, the draft genomes of *Salmonella* Typhimurium isolates. The outermost ring is the CDSs (encoding sequences), represented by the corresponding colored arrows.

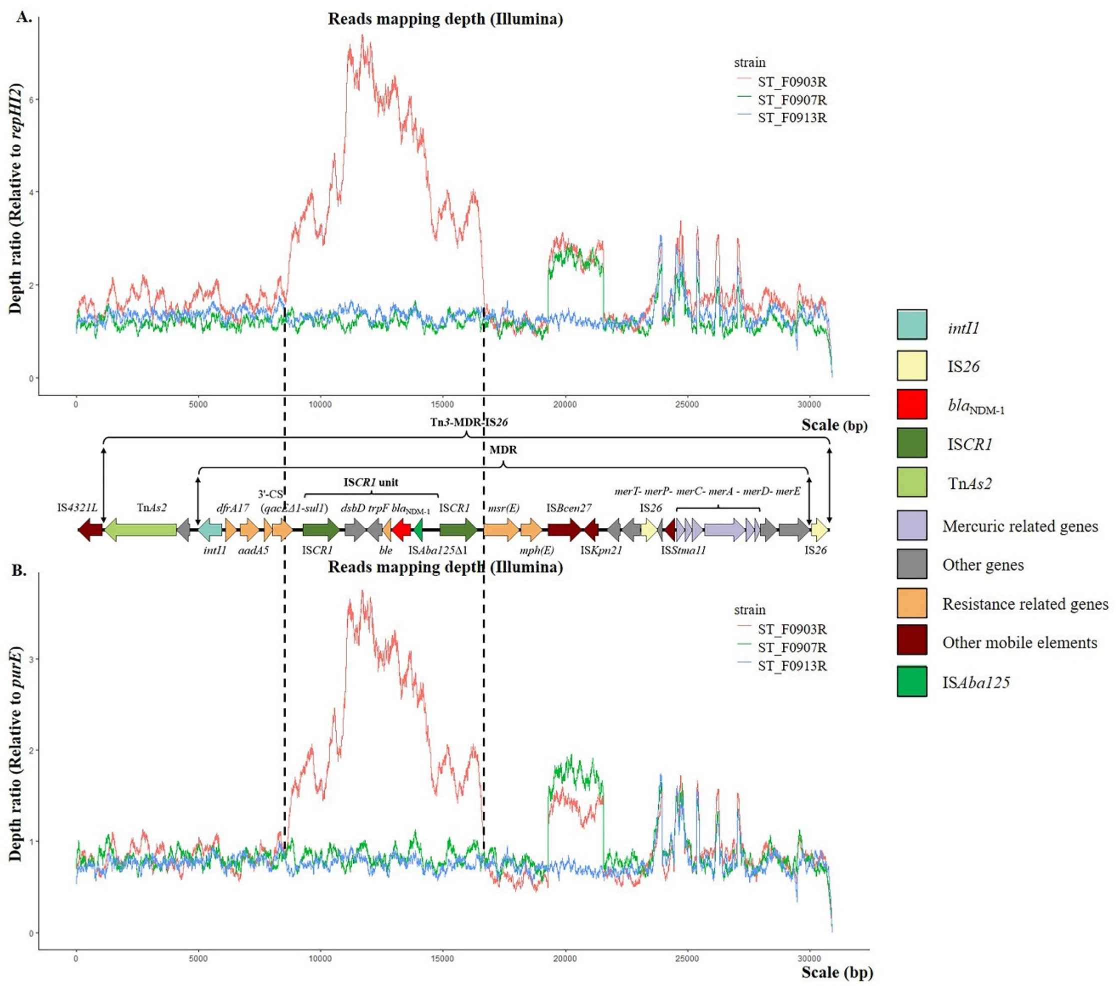

Supplementary Figure S3. Illumina sequencing reads mapping depth ratio of the Tn*As2*-MDR-IS*26* region relative to the *repHI2* and *purE* genes.
